# DNA-Protected Silver Clusters for Nanophotonics

**DOI:** 10.3390/nano5010180

**Published:** 2015-02-12

**Authors:** Elisabeth Gwinn, Danielle Schultz, Stacy M. Copp, Steven Swasey

**Affiliations:** 1 Department of Physics, The University of California, Santa Barbara, Santa Barbara, CA 93106, USA; E-Mail: shiffler@physics.ucsb.edu; 2 Department of Chemistry and Biochemistry, The University of California, Santa Barbara, Santa Barbara, CA 93106, USA; E-Mails: dschultz@chem.ucsb.edu (D.S.); sswasey@chem.ucsb.edu (S.S.)

**Keywords:** ligand-protected metal clusters, DNA templates, silver cluster, machine learning, DNA nanotechnology, fluorescence

## Abstract

DNA-protected silver clusters (Ag_N_-DNA) possess unique fluorescence properties that depend on the specific DNA template that stabilizes the cluster. They exhibit peak emission wavelengths that range across the visible and near-IR spectrum. This wide color palette, combined with low toxicity, high fluorescence quantum yields of some clusters, low synthesis costs, small cluster sizes and compatibility with DNA are enabling many applications that employ Ag_N_-DNA. Here we review what is known about the underlying composition and structure of Ag_N_-DNA, and how these relate to the optical properties of these fascinating, hybrid biomolecule-metal cluster nanomaterials. We place Ag_N_-DNA in the general context of ligand-stabilized metal clusters and compare their properties to those of other noble metal clusters stabilized by small molecule ligands. The methods used to isolate pure Ag_N_-DNA for analysis of composition and for studies of solution and single-emitter optical properties are discussed. We give a brief overview of structurally sensitive chiroptical studies, both theoretical and experimental, and review experiments on bringing silver clusters of distinct size and color into nanoscale DNA assemblies. Progress towards using DNA scaffolds to assemble multi-cluster arrays is also reviewed.

## 1. Introduction

**Figure 1:**
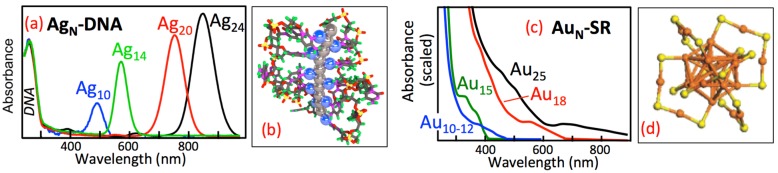
Experimental absorbance spectra are strikingly different for ligand-protected clusters composed of the same number of metal atoms but with different shapes and type of metal. (**a**) Ag_N_ clusters protected by different DNA template strands each display a single, narrow absorbance transition beyond the 260 nm DNA peak [14]; (**b**) A proposed Ag_N_-DNA structure [14], whose neutral cluster core (gray) is held by base-bound Ag^+^ (blue). The depicted *rod* cluster shape reproduces the striking spectral purity and size-dependent colors that are characteristic of Ag_N_-DNA; (**c**) The broad spectra of Au_N_-SR clusters protected by thiolate (SR) ligands reflect their *globular* cluster shape and Au composition. (Adapted from reference [15] and reprinted with permission); (**d**) The globular core of Au_N_-SR is protected by SR ligands that incorporate additional Au atoms. Orange: Au. Yellow: S. (R groups not shown). (Adapted with permission from reference [16]).

In this article, we review the properties of a compelling new class of nanoscale optical element: fluorescent silver clusters that are stabilized by single-stranded (*ss*) “binding pockets” in oligonucleotide strands and strand assemblies. Such DNA-stabilized Ag clusters (Ag_N_-DNA), first reported by Petty [1], are now emerging in applications that range from biological imaging [2], to molecular logic schemes [3] and strand-exchange on-off switches [4], to sensors for single base mutations [5,6,7], microRNAs [8], and DNA target strands [9]. These applications, recently reviewed in [10], all rely on the special sensitivity of fluorescence color and intensity to the specific nucleic acid environment of the silver cluster. Here we will focus on what our group has learned about the underlying composition and structure of pure Ag_N_-DNA that lead to these fascinating properties (Figure 1a,b) and the prospects for realizing metal cluster nanophotonics on DNA scaffolds that self-assemble by Watson-Crick pairing of synthetic DNA strands, such as DNA origami [11], tile-based assemblies [12], and nanoscale three-dimensional shapes [13].

Several groups have already demonstrated intriguing optical phenomena from the interaction of nanoscale optical elements arranged at close proximity on DNA scaffolds, including fluorescent dyes, metal nanoparticles and semiconductor quantum dots [17,18,19,20]. Due to their unique combination of metallic and molecular attributes, ligand-protected metal clusters of up to some tens of atoms in size are beginning to be examined as a distinct class of nanophotonic element. These clusters can exhibit metal-like optical response, due to the collective excitations of delocalized valence electrons, and molecule-like high fluorescence quantum yields, related to the sparse density of states in the cluster regime.

Ag_N_-DNA exemplify the special properties of metal clusters [14] and are especially exciting for nanophotonics because they integrate naturally with DNA assemblies [21] and because the DNA template can be varied to produce fluorescent silver clusters at blue-green through infrared wavelengths (Figure 1a). While atomic-level structures for Ag_N_-DNA are still not established, recent work reviewed here suggests that the selection of particular fluorescence colors by the DNA template arises largely from cluster size selection, with additional color tuning by the specific base and silver cation environment.

## 2. Results and Discussion

Our initial work on Ag_N_-DNA established the sensitivity of silver cluster fluorescence to the sequence and secondary structure of the host DNA [21]. Using select DNA oligomers, we found that visibly fluorescent silver clusters form preferentially in single-stranded (*ss*) regions of DNA hosts, rather than on double-stranded (*ds*) regions. This selectivity opened the possibility of precisely positioning silver clusters on *ss*DNA extrusions from *ds*DNA scaffolds. Additionally we used hairpin DNA templates, with *ds* stems and *ss* loops in their native state, to show that both G-and C-rich regions within the *ss* segment favor formation of fluorescent clusters, but runs of several consecutive T or A bases suppress formation of fluorescent clusters [21]. The sensitivity of silver cluster color to changes in sequence has been confirmed and expanded upon by others [22,23,24], and shown by us to extend to Ag_N_-RNAs [25].

Silver clusters formed on different oligonucleotide templates display tremendous variation in emission wavelengths (~400–800 nm), as summarized in [26], as well as widely varying quantum yields [27]. The strong variation in cluster properties with the specific details of host strands is an exciting aspect of Ag_N_-DNA that is beginning to be addressed systematically, as discussed below. However the challenge of isolating pure Ag_N_-DNA has impeded understanding of how optical properties relate to the silver content, as opposed to the base composition of the DNA template.

Ag_N_-DNA can be synthesized quite simply by borohydride reduction of solutions of Ag^+^ mixed with DNA template strands that have sequences selected to produce fluorescent solutions. This facile synthesis procedure typically produces heterogeneous solutions of many different Ag_N_-DNA species, with different numbers *N* of silver atoms attached to the DNA, as well as different numbers *n_s_* of the DNA oligomers held together by silver atoms [28]. Typically most of the silver-bearing DNA products are non-fluorescent (“dark”), with the fluorescent Ag_N_-DNA comprising only a few to some tens of percent of all silver-DNA products. This heterogeneity produced confusion in the early literature regarding the silver content, *N*, in fluorescent Ag_N_-DNA, with claims of quite small *N* = 2–3 from studies of impure solutions [21,23,29]. In contrast, in studies of pure material *N* = 10 is the smallest silver content identified to date for a fluorescent Ag_N_-DNA [14,28].

### 2.1. Isolation of Pure Ag_N_-DNA and Identification of Composition

#### 2.1.1. HPLC Separation of Ag_N_-DNA and Sizing by in-Line Mass Spectrometry

**Figure 2:**
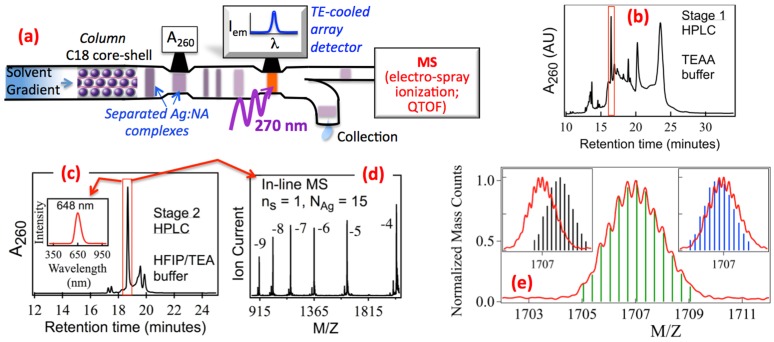
(**a**) Schematic of the HPLC-mass spectrometry (MS) system used to determine composition and optical properties of pure Ag_N_-DNA [14,28]; (**b**) Absorbance chromatogram, A_260_, from the 1st stage of purification by HPLC, with TEAA/TEA buffer, shows multiple peaks corresponding to many different Ag-bearing DNA species in the as-synthesized solution. The boxed peak is for the red fluorescent Ag_N_-DNA; (**c**) A_260_ chromatogram for the 2nd HPLC stage, with HFIP/TEA buffer, which separates out remaining impurities. This enables inline MS (**d**) to identify the red emitter as an Ag_15_-DNA containing just one DNA strand (*n_s_* = 1); (**e**) We separately identify M and Z by resolving the isotope peak envelope in high resolution MS. Alignment of measured (red) and calculated isotope patterns (bars) determines the total (*N*), neutral (*N*_0_) and cationic (*N_+_*) Ag content in Ag_N_-DNA, where the total Ag content *N = N*_0_ *+ N_+_*. (Adapted with permission from references [14,28]).

Figure 2a schematically illustrates the system we use to isolate pure Ag_N_-DNA for identification of composition and optical properties [28]. The as-synthesized, impure samples are injected into a core-shell C18 column for reverse-phase, ion pair (IP) HPLC. Because column affinities depend on the DNA conformation and composition, under optimized conditions different species of Ag_N_-DNA desorb from the column at different solvent compositions, resulting in fully or partly purified “plugs” concentrated with a particular Ag_N_-DNA. The plugs travel past an absorbance detector set to 260 nm to monitor passage of all DNA-containing material, and then past a fluorescence array detector. This method of HPLC separation is effective for more robust Ag_N_-DNA; however in several cases of strands known to produce fluorescent solutions, we have found that the Ag_N_-DNA responsible for fluorescence are too fragile to survive HPLC under the solution conditions we have employed [28].

Composition is analyzed by coupling into the electrospray ionization (ESI) source of a high resolution mass spectrometer (MS). In the ideal case of complete separation, only one species is detected in MS, identifying the composition of the Ag_N_-DNA whose properties were measured by the upstream optical detectors.

Figure 2b shows the 260 nm absorbance, A_260_, plotted *versus* time for an HPLC separation of the unpurified Ag-DNA solution formed on a typical 22-base host strand, T_2_CGC_6_GC_4_AG_2_CGT_2_, selected because it forms a nearly *spectrally* pure, red fluorescent solution [28]. However, having a single dominant *emission* peak does not indicate that the corresponding Ag_N_-DNA is the majority product in solution. In fact, as shown by the absorbance chromatogram in Figure 2b, the fluorescent Ag_N_-DNA comprises just a small fraction of the many dark Ag-DNA products formed by the synthesis. Here, the time axis corresponds to increasing methanol concentration in the water-methanol HPLC running buffer, which contains triethyl ammonium acetate (TEAA)/triethyl amine (TEA) as the ion pairing (IP) buffer. (An IP buffer is necessary for solvent-dependent retention to the C18 column.)

The many chromatogram peaks in Figure 2b each correspond to a different Ag_N_-DNA species. The red boxed peak marks the *fluorescent* Ag_N_-DNA. To identify composition, we collected the eluent plug corresponding to this peak and reinjected this partially purified material into the HPLC system for a second round of purification using a different IP system, 1,1,1,3,3,3 hexafluoro-2-propanol (HFIP)/triethylamine (TEA). Use of HFIP/TEA is crucial because it greatly enhances electrospray ionization rates of DNA relative to TEAA [30,31], providing sufficient sensitivity for small analyte quantities; and because the column retention has a different dependence on the DNA composition, which causes residual impurities from the 1st round of HPLC purification to separate (Figure 2c). For this example case, in the 2nd round HFIP/TEA separation the in-line ESI-MS shows various charge states of a *single* species with 15 Ag atoms attached to the DNA strand, identifying this red emitter as an Ag_15_-DNA (Figure 2d).

In addition to such strand monomer Ag_N_-DNA with *N* silver atoms on *n_s_* = 1 DNA strand, we have also identified strand dimer products where *n_s_* = 2 copies of the strand are glued together by the silver content [14,26,28]. Formation of fluorescent, strand dimer Ag_N_-DNA appears to dominate for short (<16 base) DNA strands [26].

Another method that has been used in attempts to determine composition of fluorescent Ag_N_-DNA is inductively coupled plasma atomic emission spectroscopy (ICP-AES) [32]. In this technique, aliquots were collected during one stage of HPLC purification, during the chromatogram peak for elution of a fluorescent product. After acid digestion, ICP-AES determines the ratio of total silver to total DNA content. If high purity is achieved in HPLC, this can be an accurate way to determine stoichiometry. However, ICP-AES provides no information on the degree of purity achieved in HPLC, and in the case of strand dimer Ag_N_-DNA can lead to a factor of two underestimation of size. Petty *et al.* discovered an IR emitting Ag_N_-DNA that forms with the template strand C_3_AC_3_AC_3_TC_3_A [32]. HPLC-MS identified this Ag_N_-DNA as a strand *dimer* (*n_s_* = 2) with *N* = 20 [28], consistent with the *N/n_s_*~10 stoichiometry found by ICP-AES, but twice as large as the composition inferred from assuming *n_s_* = 1. HPLC-MS also revealed a *non*-fluorescent product composed of just one DNA strand and 10 silver atoms: the same 10Ag/DNA strand stoichiometry as the fluorescent product, but with half the mass [28]. This dark Ag_10_-DNA had a substantially earlier column elution time than the fluorescent, strand dimer Ag_20_-DNA, consistent with the smaller DNA content [28].

Subsequent studies of other IR-emitting Ag_N_-DNA have also found *N*~20, in some cases with the silver content gluing two DNA strands together [33,34]. These papers [33,34] expressed concern that the electrospray ionization process might cause aggregation of Ag_N_-DNA. We view this as unrealistic given the pronounced physical differences between increasing the concentration of analytes in an equilibrium solution and the non-equilibrium Coulomb explosion mechanism for desolvation and ionization in ESI-MS, which is routinely used to generate and size individual ions of large biomolecules (such as proteins with molecular weights 10–20 times larger than those of Ag_N_-DNA), despite the propensity for aggregation of such large molecules in equilibrium solutions.

A third technique that has been used to estimate stoichiometry of fluorescent Ag_N_-DNA that are present in unpurified solutions is related to the “Job plot” analysis [35]. The Job plot obtains binding stoichiometry graphically from the dependence of product yields on the relative concentrations of two species that bind to form a complex, at fixed total concentration, and is known to be valid for certain limiting cases of binding reactions [35]. Studies of unpurified, red-emitting silver-DNA solutions formed on partial *ds*DNA duplexes with abasic sites used this indirect method to infer a total silver content, *N,* of approximately 2 silver atoms per DNA duplex [36]. This is a much smaller size regime than found in the above studies of purified materials, and might indicate a qualitatively different type of emissive structure; however due to use of just one total concentration, the applicability of the assumptions that underlie Job plot analyses was not tested [35].

In summary, most prior work on Ag_N_-DNA have employed unpurified solutions, which typically contain a mixture of many silver-bearing DNA products in which the fluorescent Ag_N_-DNA is a minor component. Often this may not be an issue; for example, in development of sensing schemes that are insensitive to silver-DNA byproducts. However for information about composition and structure, conclusions derived from studies of unpurified solutions [37] may be of questionable relevance.

#### 2.1.2. High Resolution ESI-MS of Ag_N_-DNA Reveals both Neutral and Cationic Silver Content

We use negative ion electrospray ionization mass spectrometry (ESI-MS) to determine how silver is incorporated into pure Ag_N_-DNA. Due to the ease of deprotonating DNA, negative ion ESI-MS is a well-established technique for determining the composition of weakly bound, noncovalent complexes with *ds*DNA or *ss*DNA in solution [38,39,40]. Use of high resolution MS (HRMS) is important because HRMS separately determines the ion mass, *M*, and charge, *Z*, rather than just the ratio *M*/*Z*. With both *M* and *Z*, we can determine how the total silver content, *N*, in Ag_N_-DNA, divides into cationic (Ag^+^) content, *N_+_*, and neutral (Ag°) content, *N*_0_. This is accomplished in HRMS by resolving the isotope peak envelope that arises from the natural variation in isotopic abundances of the elements.

Figure 2e shows an example for a particular, pure Ag_N_-DNA [14]. The finger pattern of peaks is the isotope envelope. The separation in *M*/*Z* between peaks must correspond to ∆*M* of precisely one atomic mass unit (amu), and thus identifies the charge state, *Z*. Here *Z* = −*en*_pr_ + *eN_+_*, *n*_pr_ is the number of protons removed from the DNA by ESI, *N_+_* is the number of attached Ag^+^ in the Ag_N_-DNA, and *e* is the fundamental charge. With the measured *Z*, the *M*/*Z* of the peaks determines *M*, where *M* = *M*_DNA_ + *m*_Ag_(*N_+_ + N*_0_) − *n*_pr_, M_DNA_ is the total mass of the unionized DNA and *m*_Ag_ is the silver atom mass in amu. To determine *N_+_* and *N*_0_, we vary *N_+_* to obtain agreement between the calculated and measured isotope peak envelope (Figure 2e).

Studies of many different Ag_N_-DNA, discussed below, have shown that they contain roughly equal numbers of Ag^+^ and Ag°. The Ag^+^ appear to act as part of the DNA base ligands that support a neutral, metallically bonded cluster.

**Figure 3:**
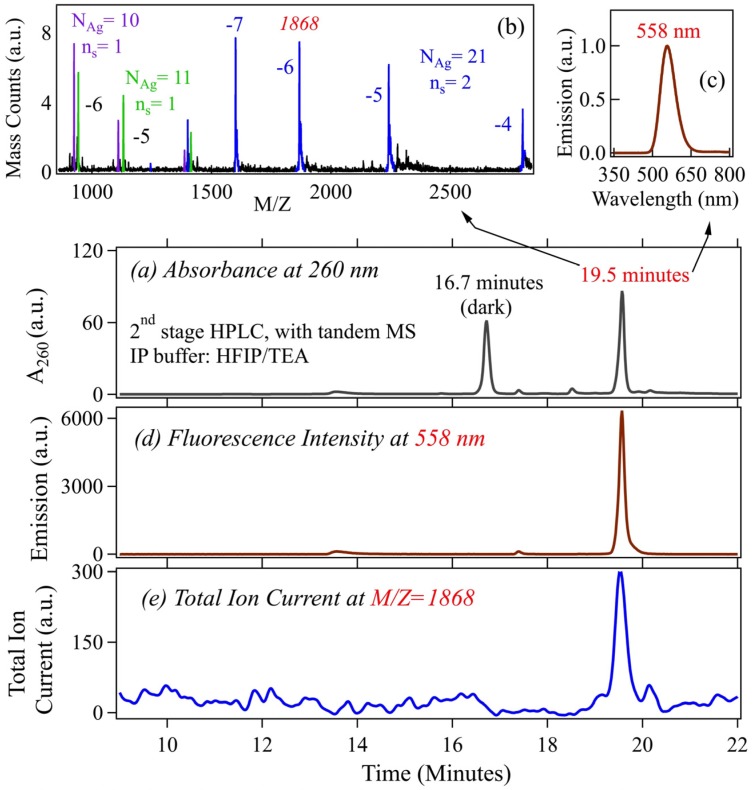
Identification of the composition of a weakly-bound strand dimer (*n_s_* = 2) Ag_N_-DNA. Chromatograms are from the 2nd stage of HPLC purification (HFIP/TEA) with inline MS. Reprinted with permission from reference [28]. (**a**) A_260_ peaks for elution of all DNA-containing species. The fluorescent Ag_N_-DNA elutes at 19.5 minutes; (**b**,**c**) MS and emission spectra corresponding to the 19.5 min A_260_ peak in (**a**); (**b**) The *Z* = −4, −5, −6 and −7 charge ladder (blue) identifies *N* = 21 as the total silver content and *n_s_* = 2 as the number of DNA strands. The additional strand monomer peaks correspond to *n_s_ =* 1 and *N* = 10 (purple) and 11 (green) fragments (as expected, the integrated fragment counts are equal within experimental error); (**d**) Fluorescence chromatogram at 558 nm; and (**e**) Mass chromatogram of the *N*_Ag_ = 21, *n_s_* = 2 IR emitter, for *M*/*Z* = 1868.

Identification of composition by ESI-HRMS can be complicated by fragmentation of delicate assemblies during ionization. For example, ESI-MS of short Watson-Crick paired DNA oligomers display peaks for the individual *ss*DNA strands as well as for the *ds*DNA duplex, due to partial disruption of hydrogen bonding in ESI. Fragmentation is also evident in MS for more delicate Ag_N_-DNA. Figure 3 shows an example that identifies a parent Ag_21_-DNA that contains *n_s_* = 2 DNA strands of the 15-base template, CGC_6_T_2_G_2_CGT [28]. ESI-MS also shows peaks for two fragments of the parent strand, an Ag_10_-DNA and an Ag_11_-DNA, each a strand monomer containing just one (*n_s_* = 1) strand. The non-overlapping HPLC retention times of strand monomer and strand dimer products establish that the monomer peaks detected in ESI-MS are generated by fragmentation, rather than being residual impurities. We infer that this Ag_21_-DNA contains two weakly bound strands, each primarily associated with *N* = 10 or 11 silver atoms. In analysis of optical properties of pure Ag_N_-DNA, discussed below, we have excluded strand dimers with this fragmentation behavior.

#### 2.1.3. Universal Excitation of Ag_N_-DNA via the Bases

Fluorescent Ag_N_-DNA are universally excited *via* the DNA bases, regardless of emission color [41]. This property is very useful for purification of Ag_N_-DNA and for rapid characterization of products formed on many different DNA hosts. In both cases we use a UV LED to excite all fluorescent Ag_N_-DNA present in solution. While the specific mechanism for the energy transfer from the initial UV-excited state to the visible (or IR) emissive state has not yet been identified, it is facilitated by the close proximity of the DNA bases and the silver clusters they bind, and may be promoted by overlap of transition energies for the bases with higher energy transitions of metal clusters, followed by decay into the lower energy emissive state.

#### 2.1.4. Native Secondary Structure of DNA and Ag_N_-DNA

Current DNA nanotechnology is built on the canonical Watson-Crick (WC) hydrogen bonding of T to A and C to G. For integration of Ag_N_-DNA into DNA nanostructures, it is important to know whether WC-paired DNA structure is maintained upon introduction of silver cations and the subsequent reduction to form silver clusters. For *ss*DNA hosts, circular dichroism studies of pure Ag_N_-DNA and of the same DNA mixed with Ag^+^, without reduction, show that Ag^+^ and Ag_N_ clusters reshape the DNA from the random coil form of the bare DNA to a more structured form [42]. One known mechanism for such structural change is the formation of Ag^+^-bridged cytosine base pairs [43], though other base-dependent pairings mediated by Ag^+^ may also be important.

A number of other Ag_N_-DNA studies have employed DNA oligomers with partial or complete WC pairing in the native DNA [10,21,36,44,45]. However, depending on the relative stabilities of WC pairing and silver-mediated interactions between the bases, the native base pairing may be disrupted by the introduction of silver cations and subsequent reduction. The assumption that native WC pairing will persist after Ag_N_ synthesis appears to hold in some cases. For 19-base, complementary C-rich and G-rich strands, the fluorescence observed for synthesis on the *individual* strands was quenched for synthesis on the *ds*DNA formed by the mixed, annealed strands, indicating that sufficient WC pairing persisted to suppress the formation of fluorescent Ag_N_-DNA [21]. Later studies of *ss*DNA homo-base strands of all T, all A, all C and all G bases showed that homobase runs of T and A do not produce fluorescent clusters at neutral pH, while homobase runs of C or G bases do template fluorescent silver clusters [25]. This is consistent with the persistence of base pairing in the G,C-rich *ds* stems of native DNA hairpins with 5 base loops, which showed only very weak emission for T and A loops, relative to the stronger fluorescence for G or C loops [21].

Other studies of native hairpins with C loops of varying length found two dominant fluorescent products, one green-emitting and one red-emitting [44]. This evidence for different structural forms of clusters formed on the same DNA host was later confirmed in electrophoretic mobility studies that found significant differences in hydrodynamic radius for the green and red emitters. The differences in diffusion constants were attributed to disruption of base pairing in the *ds* stem by the incorporation of silver to form the green emitter [45]. The necks of hairpin loops [21,44,45] and mismatches and abasic sites [36,46] in *ds*DNA provide lesions through which Ag^+^ can potentially invade to form fluorescent clusters, despite primarily *ds*DNA surroundings. Even in fully base-paired, duplex DNA, fluorescent products have been observed [46], indicating the disruption of WC pairing by silver incorporation. Successful prediction of whether a region of planned WC pairing will in fact retain native structure after silver cluster synthesis will require a better understanding of the base-specific interactions of silver with DNA.

### 2.2. Optical Properties of Pure Ag_N_-DNA and Comparison to Other Ligand-Stabilized Metal Clusters

#### 2.2.1. Sensitivity of Metal Cluster Optical Absorbance Spectra to Cluster Shape, Size and Composition

Metal *clusters* [47] are distinguished from metal *complexes* by the metal-metal bonding in clusters, which delocalizes the valence electron density over the entire cluster. Metal clusters are distinguished from metal *nanoparticles* by the small physical sizes of clusters, on the order of up some tens of atoms, corresponding to dimensions up to a few Fermi wavelengths. Because bare metal clusters agglomerate on contact, for use in solution or solid state they must be stabilized by a surface covering of protecting ligands. Most studied are small molecule ligands that cap nearly spherical, “globular”-shaped metal clusters. A well-studied example is the case of Au clusters protected by thiolate (SR) ligands (Figure 1c,d) [15,16,48].

DNA provides the protective ligand environment for Ag_N_-DNA, which are remarkable for their unique combination of metallic and molecular attributes, with metal-like behavior of their optical spectra yet molecule-like fluorescence quantum yields that can approach 100% [14]. For much larger metal nanoparticles with *N* >~10^2^–10^3^ atoms, the plasmon resonances that dominate optical spectra arise from the collective response of all the valence electrons to electromagnetic fields, which are well known to lie at energies sensitive to the shape of the metal nanoparticle [49].

**Figure 4:**
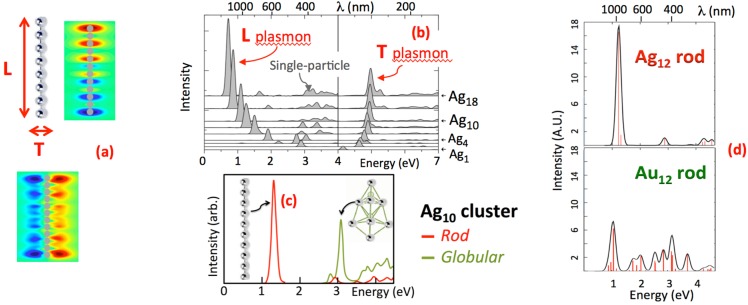
Sensitivity of cluster absorbance spectra to the cluster shape, size and composition. (**a**) An atomic silver rod, with the 2 types of collective excitations illustrated by their electron density profiles (color). Valence electrons slosh longitudinal to the rod in the “L” plasmon, and transverse to the rod in the “T” plasmon; (**b**) These L and T collective excitations dominate the optical absorbance spectra of Ag rods. Much weaker transitions are single particle-like. (Adapted with permission from reference [50]); (**c**) When the same number of Ag atoms is rearranged from a *rod* (red) into a *globule* (green), the absorbance shifts to higher energy and becomes more scattered. (Adapted with permission from reference [51]); (**d**) Calculated absorbance spectra of Ag_12_ (top) and Au_12_ (bottom) *rod* clusters. The congested spectrum for Au_12_ arises from mixing of valence electron and *d* orbital transitions. (Adapted with permission from reference [53]).

At the small sizes of metal clusters, optical transitions can still retain plasmonic (collective) character, depending on cluster composition, shape and size. In the cluster size regime, a *rod* shape uniquely concentrates the absorbance spectrum into strong collective transitions. This is illustrated in Figure 4a,b in calculated results for atomic chains of silver atoms [50]. The longitudinal “L” collective excitation of valence electrons that oscillate parallel to the rod (Figure 4a) has exceptional color tunability by rod length (Figure 4b), that strikingly resembles the Ag_N_-DNA data in Figure 1 a,b. In contrast, the weaker “T” plasmon at higher energy, with valence electrons oscillating perpendicular to the rod (Figure 4a,b), shifts relatively little with cluster size. Additional, much smaller peaks have primarily single-electron (rather than collective) character.

To display the profound effect of cluster shape, Figure 4c compares the calculated absorbance spectra of rod (red) and globular (green) Ag_10_ clusters. In the globule, scattered transitions at higher energy replace the rod’s concentrated “L” plasmon [51]. Data on globular silver clusters stabilized by *p*-MBA ligands exhibit a qualitatively similar forest of many absorbance peaks, rising above a broad background [52].

In addition to cluster shape, the choice of metal also has a profound effect on the optical spectra of clusters. In gold, *d* orbital transitions extend to lower energies than in silver. The greater mixing with valence electron transitions in gold produces congested spectra with weak absorbance peaks, as shown in calculations for Ag_12_ and Au_12_ *rods* in Figure 4d [53]. In addition to producing more complex optical spectra, the stronger *d* orbital mixing in gold may also contribute to lower fluorescence quantum yields of gold clusters, below 10% for Au_N_-SR [54], in comparison to values above 90% reported for specific Ag_N_-DNA [14].

#### 2.2.2. Evidence for Rod-Shaped Clusters in Fluorescent Ag_N_-DNA

**Figure 5:**
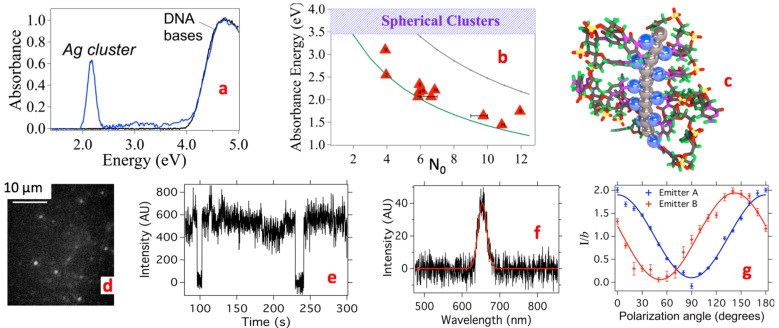
Optical properties of pure Ag_N_-DNA. (Adapted with permission from references [14,55]). (**a**) Fluorescent Ag_N_-DNA show a single absorbance peak (blue) at energies below the DNA peak. Black: DNA alone; (**b**) Peak absorbance energies of fluorescent Ag_N_-DNA (red) follow the same trend as predicted for Ag cluster rods with 1-atom cross section (green curve) [14]. Blue shading: Range of main absorbance peaks for same-size globular clusters. Gray curve: predictions for a thicker Ag cluster rod [53]; (**c**) Another example rod structure that is consistent with optical data and the measured *N*_0_ and *N*_+_. The Ag° rod (gray) is shown attached to DNA bases via Ag^+^ (blue) [14]; (**d**–**g**) Single Ag_15_-DNA at 2K (excited at 590 nm) [55]; (**d**) Wide field image shows bright spots from individual Ag_15_-DNA; (**e**) Observation of 1-step blinking between bright and dark states confirms emission from a single Ag_15_-DNA; (**f**) Spectra for individual Ag_15_-DNA remain spectrally broad at 2K, consistent with collective transitions [55]; (**g**) Dependence of emission on polarization for 2 individual Ag_15_-DNA. The high modulation index is expected for rod clusters [58].

If Ag_N_-DNA contained globular silver clusters, their absorbance spectra should show multiple peaks in the near-UV to blue, as exemplified by the green curve in Figure 4c. But instead we find that spectra of solutions of pure, fluorescent Ag_N_-DNA have a single, dominant absorbance peak (Figure 5a) at visible to near-IR wavelengths. This peak shifts to lower energy/longer wavelength with increasing neutral cluster size, *N*_0_ (Figure 1a, Figure 4b). This is the behavior predicted for rod-shaped clusters [50,53]. (Figure 5b, green line).

In cryogenic spectroscopy of individual Ag_N_-DNA emitters (Figure 4f), we found broad emission lines even at 2K (−271 °C) [55], in contrast to the dramatic line narrowing typical of deep-cooled, single organic molecules. This qualitative difference from conventional molecular behavior is another metal-like aspect of clusters, expected to arise from dephasing processes of collective excitations [56,57]. Our cryogenic microscopy also found a strong polarization dependence of individual Ag_N_-DNA, as expected for cluster rods (Figure 4g) [58].

#### 2.2.3. Magic Colors from Magic Number Cluster Sizes in Ag_N_-DNA

DNA-stabilized silver clusters are remarkable for the selection of fluorescence color by the sequence of the stabilizing DNA oligomer. Yet despite a growing number of applications that exploit this property, large-scale studies to probe the origins of Ag_N_-DNA color, and whether certain colors occur more frequently than others, have been lacking. We examined a set of 684 randomly chosen 10-base DNA oligomers to address these questions [26]. Rather than a flat distribution, we found that specific color bands dominate. Cluster size data indicate that these “magic colors” originate from the existence of magic number sizes for Ag_N_-DNA that are different from the magic number sizes characteristic of globular gold clusters stabilized by small-molecule ligands.

Cluster physics predicts enhanced abundances at certain “magic numbers” of metal atoms that are bonded together in a connected cluster, due to enhanced stabilities from electronic shell closings at these special sizes [47]. To investigate the possibility of magic numbers, and associated “magic colors”, in Ag_N_-DNA, we used robotic synthesis in a well plate format that enabled rapid read-out of emission wavelengths using a well-plate fluorimeter, with UV light exciting all fluorescent Ag_N_-DNA present in each well [26]. Of the 684 random 10-base strands, ~25% produced fluorescence with just one emission peak and a narrow enough peak width to indicate a single dominant type of fluorescent Ag_N_-DNA [26].

**Figure 6:**
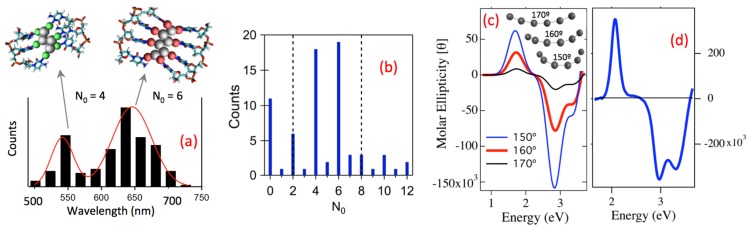
Magic colors and magic number neutral cluster sizes in (**a**,**b**) Ag_N_-DNA and chiroptical properties of pure (**c**,**d**) Ag_N_-DNA. (Adapted with permission from references [26,42]). (**a**) Fluorescence colors measured for Ag_N_-DNA formed on many different DNA templates exhibit “magic colors”: high green abundances near 540 nm and high red abundances near 630 nm [26]; (**b**) *N*_0_ = 4 and 6 are magic (highly abundant) cluster sizes across all Ag_N_-DNA with composition determined by ESI-MS [26]. Dashed lines: spherical “superatom” magic numbers 2 and 8 are ***not*** magic for Ag_N_-DNA, indicating non-spherical (rod) shapes for the silver clusters; (**c**) Calculated circular dichroism spectra for bare, chiral Ag cluster rods show a consistent pattern of positive and negative peaks for different curvatures [42]; (**d**) CD data on pure Ag_N_-DNA show the same peak pattern as theory [42].

These peak wavelengths exhibited a bimodal color distribution with enhanced (“magic”) abundances of green-emitting Ag_N_-DNA near 540 nm and red emitting Ag_N_-DNA near 630 nm (Figure 6a; the plate reader’s detector sensitivity at near-IR wavelengths was too poor to examine the possibility of magic color bands beyond ~750 nm) [26].

To investigate the origin for these magic color bands, we used the system in Figure 2a to identify Ag_N_-DNA products formed on many different strands. We found high abundances at “magic” *N*_0_ = 4 (Figure 6b), which correspond predominantly to green magic colors, and also high abundances at magic *N*_0_ = 6, which correspond predominantly to red magic colors. In contrast, numbers of silver cations, Ag^+^, spread widely; e.g., *N*_0_ = 6 clusters had *N_+_* = 6–10. Thus, neutral silver content, *N*_0_, is magic, and cationic silver content, *N*_+_, is not.

The known structures of ligand-stabilized Au clusters provide insight into why *N*_0_ is magic, but total *N* = *N*_+_ + *N*_0_ is not [48,59]. In globular Au_N_-SR, total gold content, *N*, is not magic because ligands incorporate some Au atoms, leaving cluster cores with smaller, magic number sizes that are predicted by the spherical “superatom” model. We infer that base-Ag^+^ complexes act as ligands, analogous to the Au in SR units.

A crucial difference is that DNA presents multiple base ligands along a line-like backbone, which apparently enforces rod cluster shapes. This suggests that a perimeter of base-attached Ag^+^ surrounds the cluster rod. Figure 5c shows a proposed type of structure compatible with this idea. While the structural details, such as specific sites and geometries of Ag^+^-base attachment, are not known, the optical data discussed above indicate that a rod-like neutral cluster core is key. Variations in *N*_+_ at fixed cluster core size, *N*_0_, presumably contribute to the widths of the magic color bands in Figure 6a, which exhibit a population spread of ~60 nm in red peak emission wavelengths, and ~30 nm for green peak emission wavelengths (Figure 6a).

For globular clusters, the spherical “superatom” model predicts 2 and 8 as the smallest magic numbers of cluster valence electrons. But spherical symmetry breaking in rod-shaped clusters is expected to result in non-spherical magic numbers, with an even-odd oscillation of stability as a function of size. Consistent with a non-spherical shape, the most prominent magic numbers of neutral Ag atoms in Ag_N_-DNA are 4 and 6 (Figure 6b), not 2 and 8 as for the spherical “superatom” model (dashed lines, Figure 6b).

#### 2.2.4. Chiroptical Properties of Pure Ag_N_-DNA

Measurement of cluster structure in Ag_N_-DNA by AFM, TEM or X-ray studies is problematic due to *N* of just 10–30 Ag atoms in large DNA surroundings. To obtain better structural understanding we turned to circular dichroism (CD) spectroscopy, due to its high conformational sensitivity [42].

Prior CD studies by others that used as-synthesized silver-DNA solutions [1,60,61], which typically contain mostly non-fluorescent silver-DNA products, could not draw meaningful conclusions on the minor, fluorescent product. We used pure fluorescent Ag_N_-DNA spanning the visible to near IR. Changes in the UV CD of pure cluster solutions showed that silver incorporation in fluorescent Ag_N_-DNA restructures DNA conformation [42]. The NIR through visible wavelength CD probes the chirality of the clusters themselves. We compared data to quantum chemical calculations carried out in the group of Christine Aikens [42]. They found that a rod of silver atoms with a chiral twist reproduces the special pattern of positive and negative dichroic peaks in the data, and agree in overall magnitude with experiment (Figure 6c,d) [42]. This transparent relation between cluster shape and optical chirality is in qualitative contrast to the case of gold clusters, where mixing with d-orbital transitions produces complex CD spectra [62] with no simple correlation to cluster structure.

#### 2.2.5. Equilibrium between Dark and Fluorescent forms of Ag_N_-DNA

A fascinating aspect of Ag_N_-DNA is the equilibrium between fluorescent and dark cluster forms with the same total silver content, N, that has been established for certain choices of DNA template [41,62]. This equilibrium can be manipulated by varying solution conditions, with potential for exploitation in diverse sensing applications, and may be the mechanistic underpinning for changes in fluorescence reported in some of the previously developed sensing schemes [10].

**Figure 7:**
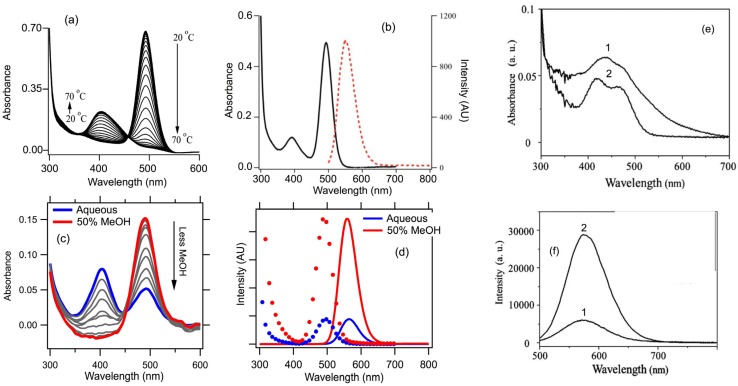
(Adapted with permission from [42,63,64]). (**a**,**b**) *Hybridization*-tuned equilibrium between dark and fluorescent forms of pure Ag_N_-DNA with 10–12 Ag atoms per DNA assembly. (**a**) Varying temperature, *T*, varies hybridization extent, producing an absorbance isosbestic point (~460 nm), that indicates an equilibrium between isomers. Peaks are at 400 nm (dark form) and 490 nm (fluorescent form) [63]; (**b**) Emission (red) and absorbance at intermediate T, with both cluster forms present. Excitation at 490 nm produces emission near 570 nm, but excitation near 400 nm produces no emission [63]; (**c**,**d**) *Solvent*-tuned equilibrium between dark and fluorescent forms of pure Ag_10_-DNA [41]; (**c**) Absorbance for MeOH fractions of 0% to 50% of the solvent, in 5% increments. The isosbestic point (near 450 nm) indicates equilibrium between two forms of the Ag_10_-DNA. Absorbance peaks at 400 nm (dark form) and 490 nm (fluorescent form) [42], just as for the hybridization-tuned equilibrium in (**a**,**b**); (**d**) Fluorescence excitation spectra (dotted) and emission spectra at 0% and 50% MeOH. The increase in intensity is due to the higher fraction of the fluorescent form [42]; (**e**,**f**) Studies of Ag-DNA and Ag-Cu-DNA made by reducing Cu^2+^ on the fluorescent Ag-DNA solution [64]; (**e**) Absorbance of unpurified solutions shows two peaks at similar wavelengths for Ag-DNA (1) and Ag-Cu-DNA (2), close to those for the dark and fluorescent cluster forms of pure Ag_N_-DNA in (**a**) and (**c**). The poorly resolved peak structure in the unpurified solutions in (**e**) indicate the presence of additional cluster species. (**f**) Emission from the unpurified Ag-DNA solution (1) increased upon reduction of Cu^2+^ (2). The 560 nm emission lies near the 560–570 nm emission peaks in the pure Ag_N_-DNAs in (**a**–**d**), suggesting that Cu^2+^ may also be controlling an equilibrium between dark and fluorescent cluster forms.

Petty and co-workers found reversible conversion between dark and fluorescent forms upon hybridization of an Ag_N_-DNA with a partially complementary strand [63]. Reversible fluorescence quenching due to thermal strand unbinding, and an associated isosbestic point in the temperature-dependent absorption spectra (Figure 7a), indicated a temperature-controlled 2-species equilibrium between a dark form with peak absorbance near 400 nm and a fluorescent form with peak absorbance near 490 nm and peak emission near 550 nm (Figure 7b). Purification by size exclusion chromatography, followed by ICP-AES, indicated a stoichiometry of approximately 11 silver atoms per strand in the dark form, and the same silver content in the 2-strand bright form.

A different means of controlling the equilibrium between between bright and dark forms of pure Ag_N_-DNA with *N* = 10 was established for an Ag_10_-DNA that formed on a particular 19-base DNA template [42]. In 50% methanol-50% aqueous solution, the absorbance spectrum exhibited a single cluster absorbance peak at 490 nm (Figure 7c) that produces an emission peak at 560 nm. Reducing the methanol fraction introduced an additional, dark form with a shorter wavelength absorbance peak near 400 nm, causing a decrease in emission intensity for excitation at 490 nm due to the decreased fraction of the fluorescent form (Figure 7d). Solvent exchange confirmed the reversibility of this bright-dark conversion, and the isosbestic point in the absorbance spectra collected for different methanol fractions indicated a 2-species equilibrium. Varying the concentration of sucrose produced similar behavior for this Ag_10_-DNA [42].

The addition of alcohols and sucrose to aqueous solution alters DNA conformation through changes in hydration [65,66]. Thus it appears that solvent-mediated changes in DNA induce structural changes in the embedded silver clusters [42]. Hybridization also alters DNA conformation. The similarities in the changes in optical properties of these Ag_N_-DNA with similar *N* = 10–11, under both solvent and hybridization-mediated control, suggest that other means of DNA structural manipulation, such as changes in ionic strength, may also induce such structural transitions of embedded clusters.

The similar stoichiometries, absorbance wavelengths of the dark and bright forms, and emission wavelengths of the bright form suggest that the different mixed-base templates used in these separate studies produced similarly structured dark and bright species. In both cases, the observed spectral switching between bright and dark forms indicates a change in bonding, perhaps due to a change in the preferred magic number cluster size that favors conversion of a larger cluster into two smaller clusters, and/or changes between neutral and cationic form of some of the silver atoms. The shorter peak absorbance wavelength of the dark form is consistent with separation of the fluorescent cluster form into smaller, dark subclusters.

#### 2.2.6. Effects of Copper and Gold Reduction on Fluorescent Silver-DNA Solutions

Changes in emission intensity have also been produced by reduction of Cu^2+^ on pre-synthesized, fluorescent Ag-DNA solutions and by simultaneous reduction of Ag^+^ and Au^3+^ on DNA [64,67]. Reduction of Cu^2+^ on fluorescent Ag-DNA solutions was reported to enhance emission near 570 nm, with almost no shift in peak wavelength. This was interpreted as evidence for incorporation of a Cu° atom into a two atom Ag° cluster, based on the most abundant peak detected in ESI-MS [64]. This is a much smaller silver content than the *N* =10–12 Ag atoms measured for pure Ag_N_-DNA with nearly the same emission wavelength (560–570) nm, as discussed above. Attempts to purify by capillary and gel electrophoresis were made in the Cu-Ag-DNA studies [64] but it is unclear whether these separations had sufficient resolution to isolate fluorescent products from dark products with similar mobilities. In our own work, we have found that electrophoresis typically fails as a purification method due to similar electrophoretic mobilities of multiple Ag_N_-DNA products.

We would expect significant shifts in wavelength, rather than the observed shift of just ~2 nm in peak emission, to result from the addition of one copper atom to a cluster of two silver atoms. Unlike Ag^+^, which bind specifically to the DNA bases, Cu^2+^ binds also to the DNA backbone [68], and may attach to the DNA at separate sites from the silver cluster. An alternative explanation for the increase in fluorescence brightness observed in [64] may be a shift in equilibrium between bright and dark silver cluster forms with the same silver content, as discussed above. Such a shift could be induced by a conformational change in the DNA from incorporation of copper at sites not directly connected to the silver cluster.

The substantial changes in fluorescence color reported for co-reduction of gold and silver cations onto DNA, relative to silver alone [67], do suggest a change in the population of fluorescent cluster sizes, perhaps reflecting the formation of Au-Ag alloy clusters. Recent work on thiolate-protected metal clusters reported the replacement of 13 gold atoms in an Au_25_ clusters with silver atoms [69]. The thiolate-protected Au_25_ cluster exhibited extremely weak fluorescence (quantum yield ~0.1%) at ~820 nm, but the thiolate-protected alloy cluster Au_12_Ag_13_ was brightly fluorescent (quantum yield ~40%) with substantially blue-shifted peak emission to 680 nm. The studies of fluorescent Au/Ag-DNA solutions claimed a composition of 3Ag° per DNA oligomer, corresponding to a ~530 nm emission peak, and a composition of 2Ag° and 1 Au°, corresponding to a ~630 nm emission peak. These assignments of composition appear to have been based on the most abundant peak detected in ESI-MS of filtered, but otherwise unpurified, solutions. Based on emission color alone, a comparison to studies of thiolate-protected Au and AuAg alloy clusters [69] and to Ag_N_-DNA purified by multiple stages of HPLC [14,28] would suggest a higher total metal content of ~10–20 Au and Ag atoms.

#### 2.2.7. The Sequence-Color Code for Ag_N_-DNA

Understanding why certain DNA sequences produce brightly fluorescent Ag_N_-DNA solutions, while other apparently similar sequences do not, is crucial for further development of these intriguing nanomaterials. This is a challenging problem due to the enormous sequence space at the strand lengths, *L*, typically used in applications: even for *L* of just 10 bases, there are over 10^6^ different sequences (4*^L = 10^*). Therefore a “hit or miss” approach is unlikely uncover specific sequences that lead to high chemical yields of Ag_N_-DNA with desired properties.

**Figure 8:**
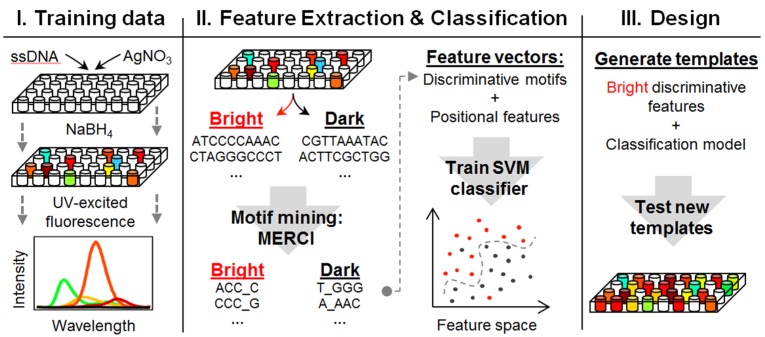
Methods used to establish that multi-base motifs are key to formation of fluorescent Ag_N_-DNA, to recognize discriminative base motifs within DNA templates and to construct new templates for solutions with increased brightness. (Adapted with permission from reference [70]). (**a**) Robotic synthesis on nearly 700 randomly generated 10-base DNA templates, and fluorescence measurement one week later, created a large, unbiased data set without unstable fluorescent products; (**b**) By running support vector machine (SVM) classifiers with different choices of feature vector, we established that multibase motifs are key to formation of fluorescent Ag_N_-DNA. A motif-miner algorithm, MERCI, was optimized to identify these discriminative motifs; (**c**) Combining the discovery of certain multi-base motifs important for determining fluorescence brightness with a simple generative algorithm, the probability of selecting DNA templates that stabilize fluorescent silver clusters was increased by a factor of >3 relative to random templates.

Our recent approach towards “cracking the code” for the sequence characteristics that govern formation of fluorescent Ag_N_-DNA is to apply machine learning algorithms to large, strategically selected data sets (Figure 8) [70]. The data is acquired by robotic synthesis and rapid array optical characterization, which make feasible the query of hundreds of distinct template strands in parallel. The underlying hypothesis is that “bright” multi-base motifs within these templates select for formation of fluorescent silver clusters, while “dark” motifs favor non-fluorescent products.

The pattern recognition algorithm we employed, the support vector machine (SVM), is a classifier that learns to separate two classes of training data, which are represented in a high-dimensional feature space, by fitting an optimal hyperplane between the two classes [71]. SVMs are widely used in bioinformatics, for example in protein-protein binding site prediction [72] and gene classification [73]. For Ag_N_-DNA, we chose the two training data classes to correspond to “bright” DNA templates that stabilize fluorescent Ag_N_-DNA and “dark” templates that do not.

We used data from randomly generated *L* = 10 base templates to train the SVM to make predictions of the probability of brightness for new, untested DNA templates. The data sets were collected one week after synthesis to eliminate unstable fluorescent products. Part of the data set was used to train the SVM, and the remainder tested the accuracy, *A*, of predictions made by the SVM. *A* is simply the fraction of test templates that the SVM correctly selects as “bright” or “dark”.

To convey information to the SVM, each bright and dark template must be represented by a “feature vector” that contains information on sequence. The most obvious choice is to use the entire template sequence, with each of the ten bases coded as an integer. However, trained SVMs using feature vectors composed of the entire sequence gave poor accuracy, *A* ≈ 60%, for bright-dark predictions. This indicates that the entire 10-base sequence is not what distinguishes bright from dark templates. By instead forming feature vectors that contain of the number of times certain multi-base motifs appear in the template, we achieved much improved accuracy, *A* > 85%, for predicting whether a new 10-base template will be bright or dark after silver cluster synthesis [70].

To identify these bright-dark discriminative motifs, we optimized a motif-miner employed in bioinformatics, MERCI [74], to recognize bright motifs that favor fluorescent Ag_N_-DNA and dark motifs that favor non-fluorescent products. The identified discriminative motifs contained 3 to 5 bases, with 4 and 5 base motifs making up 98% of those identified by MERCI [70]. Thus it appears that motifs of 4–5 base length are sufficient to define Ag_N_ clusters with the requisite structure to emit at wavelengths within the detection bandwidth of our well plate reader. Several of the identified bright motifs contain consecutive C bases and/or consecutive G bases, consistent with previous findings of fluorescent Ag_N_-DNA formed on C- and G-rich templates. We find that A bases are also common in bright motifs (the complete list of identified bright and dark motifs is given in reference [70]). Because consecutive runs of A bases were previously found not to produce fluorescent clusters [19,27], the presence of *A* bases in bright motifs suggests that having a C or G base flanking an A base may enable incorporation of silver cations in favorable modes for formation of fluorescent clusters upon reduction.

Aside from motif composition, the *number* of bright motifs required to stabilize an emissive Ag cluster is important for template design strategies. In studies of composition, we have found that for template strands with 16 or fewer bases, *two* copies of the same strand simultaneously stabilized the clusters. This implies that at least two bright motifs are required. In longer templates, this cluster “sandwiching” between bright motifs can be achieved by folding the strand around the cluster. With shorter templates, stiffness at length scales below the ~2 nm persistence length of *ss*DNA may preclude such folding, so that clusters instead engage multiple bright motifs by simultaneously attaching to two strands [70].

To create new DNA templates for bright Ag_N_-DNA solutions, we used a simple generative algorithm that biases the template contents by including brightness-weighted discriminative motifs [70]. We classified each of the newly generated templates as bright or dark using our previously trained SVM and experimentally tested the effectiveness of this motif-based design method for the 374 template sequences to which the SVM assigned the highest brightness probabilities. The average fluorescence brightness of Ag_N_-DNA solutions synthesized with this designed template set was much brighter, by a factor of >3 at one week after synthesis, relative to the random template set used to train the SVM. This inclusion of greater numbers of “bright” base motifs also red-shifted the Ag_N_-DNA color distribution of the designed templates relative to the random templates. It appears that the higher average number of bright motifs in the designed templates increased the numbers of Ag^+^ incorporated before reduction, causing an increase in the average silver cluster size formed by reduction and thus longer fluorescence wavelength. We expect that a more sophisticated generative algorithm, incorporating sparse dark motifs to limit byproducts, may further increase chemical yields of fluorescent products, and further anticipate that the motif miner-SVM approach can be generalized to prediction of templates that select for Ag_N_-DNA color.

#### 2.2.8. Stabilities of Ag_N_-DNA

Just as Ag_N_-DNA display widely varying colors and fluorescence quantum yields, their solution stabilities also vary widely for reasons that are not well understood. Temporal stability ranges from very poor, with fluorescence decaying over a matter of hours for storage at room temperature [75], to excellent: emission of a 530 nm emitting Ag_11_-DNA [44] has persisted over several years in our labs, and a 615 nm emitting Ag_N_-DNA with stability over at least one year of storage has also been reported [60]. For Ag_N_-DNA that are stable enough to isolate by HPLC, we find that pure solutions are typically stable over weeks to months for storage at 10 °C at micromolar concentrations.

Another important issue for many applications is photostability. Early studies of fluorescent, ~700 nm emitting Ag_N_-DNA that were immobilized in polyvinyl alcohol films (PVA) found high photostability, superior to cyanine dyes [29]. For many biomolecular applications, solution photostability is relevant rather than behavior in PVA. Solution photostability of Ag_N_-DNA is poor in some cases, with wide variations in photobleaching rates amongst fluorescent solutions formed on different DNA templates [75,76]. These photostability studies proposed a redox-controlled mechanism for the photobleaching of a red-emitting Ag_N_-DNA that simultaneously enhanced fluorescence from a green-emitting Ag_N_-DNA. A redox reaction was also indicated by the conversion of green-emitting solutions back to the red form by addition of NaBH_4_ [76]. Earlier studies had identified the green emitter as an Ag_11_-DNA and the red emitter as an Ag_13_-DNA [44] with a smaller hydrodynamic radius [45], so the interconversion changes DNA conformation in addition to adding or removing silver. The two silver atoms “lost” by the Ag_13_-DNA to form the Ag_11_-DNA may transfer to other strands in solution. In general, changes in solution conditions that alter fluorescence color presumably signal changes in cluster size by inter- or inter-strand transfer of silver content.

#### 2.2.9. Electronic Properties of the Bases and Ag_N_-DNA

The base-specific interactions of silver with the DNA template and competition of cluster formation with base pairing, and with agglomeration to form larger metal particles, presumably all underlie the wide variation in Ag_N_-DNA properties found for seemingly minor variations in DNA template sequence. Early studies of Ag_N_-DNA and Ag_N_-RNA found that fluorescent clusters formed on strands of all C or all G bases, but not on strands of all T or all A bases at neutral pH [28]. The persistence of Ag^+^ content as roughly half the total silver in pure Ag_N_-DNA suggests that favorable cationic interactions with the bases are key. In the case of adenine homopolymer DNA strands, the absence of fluorescent products is puzzling given the presence of endocyclic nitrogen atoms in A, as in C and G but not T, and suggests that the specific geometry of Ag^+^-adenine binding is incompatible with the formation of the Ag°-Ag° bonds that are integral to clusters.

It appears that the different binding interactions of silver cations with the different bases lead to multi-base motifs [70] that favor selection of specific “magic” neutral silver clusters sizes upon reduction [26]. The roughly 60 nm spread in emission wavelength of clusters containing six Ag° shows that there are additional color-tuning mechanisms at play. Shape variations, such as curvature of rod-shaped clusters [26] and or variation in dihedral Ag°-Ag°-Ag° bond angles [77], may contribute. Additionally, the specific base content in the cluster-stabilizing motif presumably also tunes color through inductive chemical interactions, in analogy to the rather weak effects on optical properties of Au_38_ clusters observed for addition of electron donating or withdrawing groups to the stabilizing ligands [78].

### 2.3. Ag_N_-DNA Assemblies

DNA nanotechnology allows precise nano-scale arrangement of optical elements such as nanoparticles and dye molecules onto scaffolds with diverse, DNA-programmed sizes and shapes [11,79]. Such DNA constructs have already demonstrated intriguing phenomena from interactions of arrayed elements, including FRET [17], optical chirality [18], and surface-enhanced Raman scattering [20]. Achieving the desired assembly properties requires stringent control over individual elements, a challenge for nanoparticles due to the difficulty of achieving precise sizes, shapes and surface morphologies [80] with DNA-compatible chemistries.

In contrast, ligand-protected metal clusters offer *atomically precise* control of optical behavior. A great advantage of metal clusters is their unique combination of metallic and molecular attributes, which can bring together strong collective excitations of valence electrons with high fluorescence quantum yields related to the sparse density of states. Developing methods for arranging metal clusters in designed nanoscale patterns is a necessary step for harnessing their potential for applications in nanotechnology.

#### 2.3.1. Bi-Color, Dual Cluster Ag_N_-DNA Assemblies

With their DNA template-tuned colors, compact sizes, biocompatibility and high fluorescence quantum yields, Ag_N_-DNA are exciting prospects for multicolor decoration of DNA assemblies. However, this requires Ag clusters of different size to retain stability when held in nanoscale proximity. DNA templates that provide high enough barriers are needed to prevent reorganization driven by the size-dependence of bare cluster free energies [47,81]. But solutions containing DNA-stabilized Ag clusters of unknown composition exhibited color and brightness changes when mixed with additional DNA strands [9]. This raises the question of whether it is possible to select templates that preserve the stability of different sized clusters when they are brought together into a single nanoscale construct.

**Figure 9:**
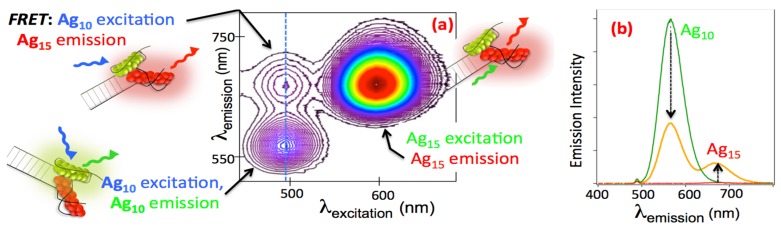
Development of dual cluster, bicolor silver cluster assemblies. Schematic to the left: Cartoons: paired cluster assemblies. Red: Ag_15_; Green: Ag_10_. (**a**) Contour map of emission intensity from the purified solution of paired clusters shows the expected peaks for direct excitation of Ag_10_ and Ag_15_, and FRET: emission from Ag_15_ due to excitation of Ag_10_, via radiationless energy transfer [82]; (**b**) Emission excited at the Ag_10_ absorbance peak. Green and (baseline) red curves are emission from separate solutions of the individual clusters. Orange: FRET of paired clusters produces emission from Ag_15_ and reduces Ag_10_ emission. The unshifted wavelengths relative to the individual clusters verify intact, stable assembly. (Adapted with permission from reference [82]).

We developed 2-color constructs as test-of-principle experiments. The templates for each cluster have appended hybridization “tails”, designed to bring red (*N* = 15) and green (*N* = 10) Ag_N_-DNA together in a “clamp” assembly (Figure 9a) [82]. Observation of fluorescence resonance energy transfer (FRET; Figure 9a,b) established that these assemblies hold the clusters at separations below 6.2 nm, the Förster radius of the Ag_10_-Ag_15_ pair [82]. Thermally-modulated FRET confirmed that the dual cluster structures disassemble and reassemble under thermal cycling [82]. The absence of spectral shifts in these dual-cluster FRET pairs, relative to the individual clusters, showed that select few-atom silver clusters of different sizes are sufficiently stable to retain structural integrity when held within a single nanoscale DNA construct.

#### 2.3.2. Ag_N_-DNA on DNA scaffolds

**Figure 10:**
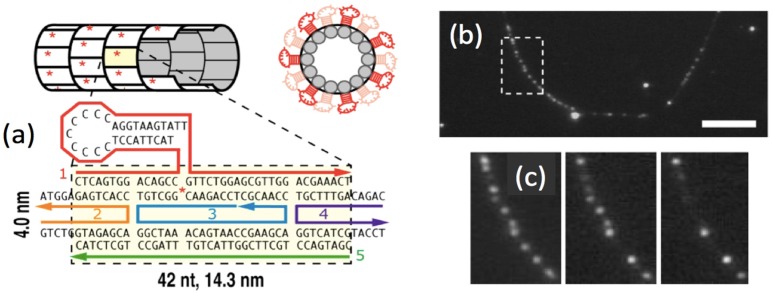
Decoration of a DNA nanotube with fluorescent Ag_N_-DNA at ~1 µm separations. (Adapted with permission from reference [83]). (**a**) Nanotube design, with a native DNA hairpin extruding from every tile; (**b**) Fluorescence microscopy of nanotubes assembled with hairpins on 0.5% of tiles. Direct synthesis onto the assembled tubes produces bright emission spots from fluorescent red silver clusters; (**c**) Photobleaching of emission spots along the linear nanotube contour enables estimation of ~45% initial occupation of hairpin protrusions by the unstable red clusters.

While dense, atomically precise arrays of Ag_N_-DNAs have not yet been realized on larger DNA scaffolds assembled from many constituent strands, there has been progress towards this goal. The preference for Ag cluster formation onto *ss*DNA [21] was exploited by O’Neill and Fygenson to synthesize Ag_N_-DNAs on DNA nanotubes with hairpin protrusions [83]. Sparse cluster decoration, with average separations of ~1 µm, enabled an estimate of ~45% initial occupation of all hairpin protrusions by unstable red fluorescent clusters (Figure 10). Shifts in emission wavelength on tubes, relative to the hairpin free in solution, may indicate formation of a slightly different mixture of red-emitting cluster sizes. Despite the lack of long-term stability, this is an encouragingly high synthesis yield of fluorescent Ag_N_-DNA products. It was achieved by using a native hairpin that produces unusually high yields of fluorescent products [44], for reasons that are not well understood. However the progress described above in realizing more stable Ag_N_-DNA, and in the emerging understanding of multi-base discriminative motifs, suggests that the need for high synthesis yields of stable Ag_N_-DNA may be within reach.

Achieving atomically precise arrays of metal clusters at the <10 nm separations afforded by DNA scaffolds will require methods that achieve ~100 times more closely spaced clusters, with monodisperse cluster sizes. For much larger metal nanoparticles coated with DNA, attachment to DNA origami was limited to edge-to-edge particle separations of ~30 nm or greater by the repulsion of the negatively charged DNA [84]. However as shown above, silver clusters can be held by DNA at separations below 10 nm, perhaps in part due to a reduction of backbone repulsion by the Ag^+^ that are incorporated into Ag_N_-DNA. Therefore we anticipate that electrostatic effects will not prevent formation of dense arrays of silver clusters on DNA scaffolds.

Fluorescent Ag_N_-DNA have also been used to decorate DNA nanowires [85] and DNA hydrogels [86], by incorporating loops of unpaired C bases into the strands that Watson-Crick pair to form these one-dimensional and three-dimensional scaffolds. Synthesis of silver clusters was performed directly onto the scaffolds, and fluorescence was observed in both of these widely differing geometries. Because synthesis typically produces abundant dark silver-DNA byproducts that occupy designed sites for fluorescent silver clusters, it is unknown what fraction of the intended scaffold sites were occupied by fluorescent Ag_N_-DNA.

## 3. Conclusions

Rod-like metal clusters were initially realized using a STM to position Au atoms on atomically flat substrates in ultra-high vacuum [87]. It appears that that silver cluster rods [14] can instead be assembled at low cost in aqueous solution by using base motifs [70] within DNA templates as cluster-protecting ligands. The fluorescence and color tunability of Ag_N_-DNA are already being exploited in novel sensing applications. Thus the atomic metal rod, a beautiful model system, is now becoming technologically relevant.

While the sensitivity of fluorescence wavelengths to the specifics of the host DNA template is an exciting aspect of Ag_N_-DNA, a coherent picture is still lacking of just how certain templates favor fluorescent Ag_N_-DNA of a given size and color. Towards achieving this understanding, we have used large array data sets and machine learning tools to establish that multi-base motifs govern the fluorescence brightness of Ag_N_-DNA solutions [70]. We separately identified sets of motifs that select for brightness and sets of motifs that discriminate against bright products. Both motif types will be important for realizing designed multi-cluster constructs.

Many basic open questions remain regarding Ag_N_-DNA. These include the factors that govern Stokes shifts and the widely-varying fluorescence quantum yields as well as the specific roles of the bases in cluster structure and color. However the work to date showing that clusters of distinct size and color retain their stability when held in nanoscale proximity [82] is promising for development of atomically precise, metal cluster nanophotonics on the versatile scaffold geometries provide by DNA nanotechnology.

## References

[1] Petty J.T., Zheng J., Hud N.V., Dickson R.M. (2004). DNA-templated Ag nanocluster formation. J. Am. Chem. Soc..

[2] Yu J., Choi S., Richards C.I., Antoku Y., Dickson R.M. (2008). Live cell surface labeling with fluorescent Ag nanocluster composites. Photochem. Photobiol..

[3] Li T., Zhang L.B., Ai J., Dong S., Wang E.K. (2011). Ion-tuned DNA/Ag fluorescent nanoclusters as versatile logic device. ACS Nano.

[4] Guo W., Yuan J., Wang E.K. (2011). Strand exchange reaction modulated fluorescence “on-off” switching of hybridized DNA duplex stabilized silver nanoclusters. Chem. Comm..

[5] Guo W.W., Yuan J.P., Dong Q.Z., Wang E.K. (2010). Highly sequence dependent formation of fluorescent silver nanoclusters in hybridized DNA duplexes for single nucleotide mutation identification. J. Am. Chem. Soc..

[6] Ma K., Cui Q.H., Liu G.Y., Wu F., Xu S.J., Shao Y. (2011). DNA abasic site-directed formation of fluorescent silver nanoclusters for selective nucleobase recognition. Nanotechnology.

[7] Yeh H.-C., Sharma J., Shih I.-M., Vu D.M., Martinez J.S., Werner J.H. (2012). A fluorescence light-up Ag nanocluster probe that discriminates single-nucleotide variants by emission color. J. Am. Chem. Soc..

[8] Yang S.W., Vosch T. (2011). Rapid detection of microRNA by a silver nanocluster DNA probe. Anal. Chem..

[9] Yeh H.-C., Sharma J., Han J.J., Martinez J.S., Werner J.H. (2010). A DNA-silver nanocluster probe that fluoresces upon hybridization. Nano Lett..

[10] Yuan Z., Chen Y.-C., Li H.-W., Chang H.-T. (2014). Fluorescent silver nanoclusters stabilized by DNA scaffolds. Chem. Comm..

[11] Rothemund P.W.K. (2006). Folding DNA to create nanoscale shapes and patterns. Nature.

[12] Winfree E., Liu F., Wenzler L.A., Seeman N.C. (1998). Design and self-assembly of two-dimensional DNA crystals. Nature.

[13] Douglas S.M., Dietz H., Liedl T., Högberg B., Graf F., Shih W.M. (2009). Self-assembly of DNA into nanoscale three-dimensional shapes. Nature.

[14] Schultz D., Gardner K., Oemrawsingh S.S.R., Markešević N., Olsson K., Debord M., Bouwmeester D., Gwinn E. (2013). Evidence for rod-shaped DNA-stabilized silver nanocluster emitters. Adv. Mater..

[15] Stamplecoskie K.G., Kamat P.V. (2014). Size-dependent excited state behavior of glutathione-capped gold clusters and their light-harvesting capacity. J. Am. Chem. Soc..

[16] Häkkinen H. (2008). Atomic and electronic structure of gold clusters: Understanding flakes, cages and superatoms from simple concepts. Chem. Soc. Rev..

[17] Stein I.H., Schüller V., Böhm P., Tinnefeld P., Liedl T. (2011). Single-molecule FRET ruler based on rigid DNA origami blocks. Chemphyschem.

[18] Kuzyk A., Schreiber R., Fan Z., Pardatscher G., Roller E.-M., Högele A., Simmel F.C., Govorov A.O., Liedl T. (2012). DNA-based self-assembly of chiral plasmonic nanostructures with tailored optical response. Nature.

[19] Shen X., Asenjo-Garcia A., Liu Q., Jiang Q., García de Abajo F.J., Liu N., Ding B. (2013). Three-dimensional plasmonic chiral tetramers assembled by DNA origami. Nano Lett..

[20] Pilo-Pais M., Watson A., Demers S., LaBean T.H., Finkelstein G. (2014). Surface-enhanced raman scattering plasmonic enhancement using DNA origami-based complex metallic nanostructures. Nano Lett..

[21] Gwinn E.G., O’Neill P., Guerrero A., Bouwmeester D., Fygenson D.K. (2008). Sequence-dependent fluorescence from DNA-hosted silver nanoclusters. Adv. Mater..

[22] Richards C.I., Choi S., Hsiang J.C., Antoku Y., Vosch T., Bongiorno A., Teng Y.L., Dickson R.M. (2008). Oligonucleotide-stabilized Ag nanocluster fluorophores. J. Am. Chem. Soc..

[23] Sengupta B., Ritchie C.M., Buckman J.G., Johnsen K.R., Goodwin P.M., Petty J.T. (2008). Base-directed formation of fluorescent silver clusters. J. Phys. Chem. C.

[24] Sengupta B., Springer K., Buckman J.G., Story S.P., Hasan Z.W., Prudowsky Z.D., Rudisill S.E., Degtyareva N.N., Petty J.T. (2009). DNA templates for fluorescent silver clusters and i-motif folding. J. Phys. Chem. C.

[25] Schultz D., Gwinn E.G. (2011). Stabilization of fluorescent silver clusters by RNA homopolymers and their DNA analogs: C,G *versus* A,T(U) dichotomy. Chem. Comm..

[26] Copp S.M., Schultz D., Swasey S., Pavlovich J., Debord M., Chiu A., Olsson K., Gwinn E. (2014). Magic numbers in DNA-stabilized fluorescent silver clusters lead to magic colors. J. Phys. Chem. Lett..

[27] Jaswinder S., Yeh H.C., Yoo H., Werner J.H., Martinez J.S. (2010). A complementary palette of fluorescent silver nanoclusters. Chem. Comm..

[28] Schultz D., Gwinn E.G. (2012). Silver atom and strand numbers in fluorescent and dark Ag:DNAs. Chem. Comm..

[29] Vosch T., Antoku Y., Hsiang J.C., Richards C.I., Gonzales J.I., Dickson R.M. (2007). Strongly emissive individual DNA-encapsulated Ag nanoclusters as single-molecule fluorophores. Proc. Nat. Acad. Sci. USA.

[30] Fountain K.J., Gilar M., Gebler J.C. (2003). Analysis of native and chemically modified oligonucleotides by tandem ion-pair reversed-phase high-performance liquid chromatography/electrospray ionization mass spectrometry. Rapid Comm. Mass Spec..

[31] Gilar M., Fountain K.J., Budman Y., Neue U.D., Yardley K.R., Rainville P.D., Russell R.J., Gebler J.C. (2002). Ion-pair reversed-phase high-performance liquid chromatography analysis of oligonucleotides: Retention prediction. J. Chromat. A.

[32] Petty J.T., Fan C.Y., Story S.P., Sengupta B., Iyer A.S., Prudowsky Z., Dickson R.M. (2010). DNA encapsulation of 10 silver atoms producing a bright, modulatable, near-infrared-emitting cluster. J. Phys. Chem. Lett..

[33] Petty J.T., Nicholson D.A., Sergev O.O., Graham S.K. (2014). Near-infrared silver cluster optically signaling oligonucleotide hybridization and assembling two DNA hosts. Anal. Chem..

[34] Petty J.T., Giri B., Miller I.C., Nicholson D.A., Sergev O.O., Banks T.M., Story S.P. (2013). Silver clusters as both chromophoric reporters and DNA ligands. Anal. Chem..

[35] Huang C.Y. (1982). Determination of the binding stoichiometry by the continuous variation method: The job plot. Meth. Enzymol..

[36] Ma K., Shao Y., Ciu Q., Wu F., Xu S., Liu G. (2012). Base-stacking determined fluorescence emission of DNA abasic site-templated silver clusters. Langmuir.

[37] Neidig M.L., Sharma J., Yeh H.-C., Martinez J.S., Conradson S.D., Shreve A.P. (2011). Ag K-edge EXAFS analysis of DNA-templated fluorescent silver nanoclusters: Insight into the structural origins of emission tuning by DNA sequence variations. J. Am. Chem. Soc..

[38] Greig M.J. (2000). Detection of oligonucleotide-ligand complexes by ESI-MS (DOLCE-MS) as a component of high throughput screening. J. Biomol. Screen..

[39] Hofstadler S., Sannes-Lowry K. (2006). Applications of ESI-MS in drug discovery: Interrogation of non-covalent complexes. Nat. Rev. Drug Discov..

[40] Rosu F., Gabelica V., Houssier C., de Pauw E. (2002). Determination of affinity, stoichiometry and sequence selectivity of minor groove binder complexes with double-stranded oligodeoxy-nucleotides by electrospray ionization mass spectrometry. Nucleic Acids Res..

[41] O’Neill P.R., Gwinn E.G., Fygenson D.K. (2011). UV Excitation of DNA-stabilized Ag-cluster fluorescence via the DNA bases. J. Phys. Chem. C.

[42] Swasey S.M., Karimova N., Aikens C.M., Schultz D.E., Simon A.J., Gwinn E.G. (2014). Chiral electronic transitions in fluorescent silver clusters stabilized by DNA. ACS Nano.

[43] Ono A., Cao S., Togashi H., Tashiro M., Fujimoto T., Machinami T., Oda S., Miyake Y., Okamoto I., Tanaka Y. (2008). Specific interactions between silver (I) ions and cytosine-cytosine pairs in DNA duplexes. Chem. Comm..

[44] O’Neill P.R., Velazquez L.R., Dunn D.G., Gwinn E.G., Fygenson D.K. (2009). Hairpins with poly-C loops stabilize four types of fluorescent Ag_n_:DNA. J. Phys. Chem. C.

[45] Driehorst T., O’Neill P.R., Goodwin P.J., Pennathur S., Fygenson D.K. (2011). Distinct conformations of DNA-stabilized fluorescent silver nanoclusters revealed by electrophoretic mobility and diffusivity measurements. Langmuir.

[46] Shah P., Rorvig-Lund A., Ben Chaabane S., Thulstrup P.W., Kjaergaard H.G., Fron E., Hofkens J., Yang S.W., Vosch T. (2012). Design aspects of bright red emissive silver nanoclusters/DNA probes for microRNA detection. ACS Nano.

[47] De Heer W.A. (1993). The physics of simple metal clusters: Experimental aspects and simple models. Rev. Mod. Phys..

[48] Walter M., Akola J., Lopez-Acevedo O., Jadzinsky P.D., Calero G., Ackerson C.J., Whetten R.L., Grönbeck H., Häkkinen H. (2008). A unified view of ligand-protected gold clusters as superatom complexes. Proc. Natl. Acad. Sci. USA.

[49] Link S., Mohamed M.B., El-Sayed M.A. (1999). Simulation of the optical absorption spectra of gold nanorods as a function of their aspect ratio and the effect of the medium dielectric constant. J. Phys. Chem. B.

[50] Yan J., Gao S. (2008). Plasmon resonances in linear atomic chains: Free-electron behavior and anisotropic screening of d electrons. Phys. Rev. B.

[51] Baishya K., Idrobo J., Öğüt S., Yang M., Jackson K., Jellinek J. (2008). Optical absorption spectra of intermediate-size silver clusters from first principles. Phys. Rev. B.

[52] Desireddy A., Conn B.E., Guo J., Yoon B., Barnett R.N., Monahan B.M., Kirschbaum K., Griffith W.P., Whetten R.L., Landman U. (2013). Ultrastable silver nanoparticles. Nature.

[53] Guidez E.B., Aikens C.M. (2012). Theoretical analysis of the optical excitation spectra of silver and gold nanowires. Nanoscale.

[54] Yu Y., Luo Z., Chevrier D.M., Leong D.T., Zhang P., Jiang D., Xie J. (2014). Identification of a highly luminescent Au_22_(SG)_18_ nanocluster. J. Am. Chem. Soc..

[55] Oemrawsingh S.S.R., Markešević N., Gwinn E.G., Eliel E.R., Bouwmeester D. (2012). Spectral properties of individual DNA-hosted silver nanoclusters at low temperatures. J. Phys. Chem. C.

[56] Lermé J., Lyon D., Kastler A. (2011). Size evolution of the surface plasmon resonance damping in silver nanoparticles : Confinement and dielectric effects. J. Phys. Chem. C.

[57] Weick G., Molina R., Weinmann D., Jalabert R. (2005). Lifetime of the first and second collective excitations in metallic nanoparticles. Phys. Rev. B.

[58] Markešević N., Oemrawsingh S.S.R., Schultz D., Gwinn E.G., Bouwmeester D. (2014). Polarization resolved measurements of individual DNA-stabilized silver clusters. Adv. Opt. Mater..

[59] Walter M., Whetten R.L., Ha H., Gro H. (2008). On the Structure of Thiolate-Protected Au 25. J. Am. Chem. Soc..

[60] Sharma J., Rocha R.C., Phipps M.L., Yeh H.C., Balatsky K.A., Vu D.M., Shreve A.P., Werner J.H., Martinez J.S. (2012). A DNA-templated fluorescent silver nanocluster with enhanced stability. Nanoscale.

[61] Lan G.Y., Chen W.Y., Chang H.T. (2011). One-pot synthesis of fluorescent oligonucleotide Ag nanoclusters for specific and sensitive detection of DNA. Biosens. Bioelectron..

[62] Dolamic I., Knoppe S., Dass A., Bürgi T. (2012). First enantioseparation and circular dichroism spectra of Au_38_ clusters protected by achiral ligands. Nat. Commun..

[63] Petty J.T., Sergev O.O., Nicholson D.A., Goodwin P.M., Giri B., McMullan D.R. (2013). A silver cluster-DNA equilibrium. Anal. Chem..

[64] Lan G.Y., Chen W.Y., Chang H.T. (2011). Characterization and application to the detection of single-stranded DNA binding protein of fluorescent DNA-templated copper/silver nanoclusters. Analyst.

[65] Son I., Shek Y.L., Dubins D.N., Chalikian T.V. (2014). Hydration changes accompanying helix-to-coil DNA transitions. J. Am. Chem. Soc..

[66] Spink C.H., Garbett N., Chaires J.B. (2007). Enthalpies of DNA melting in the presence of osmolytes. Biophys. Chem..

[67] Chen W.-Y., Lan G.-Y., Chang H.-T. (2011). Use of fluorescent DNA-templated gold/silver nanoclusters for the detection of sulfide ions. Anal. Chem..

[68] Berti L., Burley G.A. (2008). Nucleic acid and nucleotide-mediated synthesis of inorganic nanoparticles. Nat. Nanotechnol..

[69] Wang S.X., Meng X.M., Das A., Li T., Song Y.B., Cao T.T., Zhu X.Y., Zhu M.Z., Jin R.C. (2014). A 200-fold quantum yield boost in the photoluminescence of silver-doped Ag(x)Au(25 − x) nanoclusters: The 13th silver atom matters. Angew. Chem. Int. Ed..

[70] Copp S.M., Bogdanov P., Debord M., Singh A., Gwinn E. (2014). Base motif recognition and design of DNA templates for fluorescent silver clusters by machine learning. Adv. Mater..

[71] Cortes C., Vapnik V. (1995). Support vector networks. Mach. Learn..

[72] Bradford J.R., Westhead D.R. (2005). Improved prediction of protein-protein binding sites using a support vector machines approach. Bioinformatics.

[73] Brown M.P.S., Grundy W.N., Lin D., Cristianini N., Sugnet C.W., Furey T.S., Ares M., Haussler D. (2000). Knowledge-based analysis of microarray gene expression by using support vector machines. Proc. Natl. Acad. Sci. USA.

[74] Vens C., Rosso M.N., Danchin E.G.J. (2011). Identifying discriminative classification-based motifs in biological sequences. Bioinformatics.

[75] Walczak S., Morishita K., Ahmed M., Liu J. (2014). Towards understanding of poly-guanine activated fluorescent silver nanoclusters. Nanotechnology.

[76] Morishita K., MacLean J.L., Liu B., Jiang H., Liu J. (2013). Correlation of photobleaching, oxidation and metal induced fluorescence quenching of DNA-templated silver nanoclusters. Nanoscale.

[77] Ramazanov R., Kononov A. (2013). Excitation spectra argue for threadlike shape of DNA-stabilized silver fluorescent clusters. J. Phys. Chem. C.

[78] Guo R., Murray R.W., Hill C., Carolina N. (2005). Substituent effects on redox potentials and optical gap energies of molecule-like Au_38_(SPhX)_24_ nanoparticles. J. Am. Chem. Soc..

[79] Yin P., Hariadi R.F., Sahu S., Choi H.M. T., Park S.H., Labean T.H., Reif J.H. (2008). Programming DNA tube circumferences. Science.

[80] Lee Y.-J., Schade N.B., Sun L., Fan J.A., Bae D.R., Mariscal M.M., Lee G., Capasso F., Sacanna S., Manoharan V.N. (2013). Ultrasmooth, highly spherical monocrystalline gold particles for precision plasmonics. ACS Nano.

[81] Guticrez M., Henglein A. (1993). Formation of colloidal silver by “push-pull” reduction of Ag^+^. J. Phys. Chem..

[82] Schultz D., Copp S.M., Markešević N., Gardner K., Oemrawsingh S.S.R., Bouwmeester D., Gwinn E. (2013). Dual-color nanoscale assemblies of structurally stable, few-atom silver clusters, as reported by fluorescence resonance energy transfer. ACS Nano.

[83] O’Neill P.R., Young K., Schiffels D., Fygenson D.K. (2012). Few-atom fluorescent silver clusters assemble at programmed sites on DNA nanotubes. Nano Lett..

[84] Pilo-Pais M., Goldberg S., Samano E., Labean T.H., Finkelstein G. (2011). Connecting the nanodots: Programmable nanofabrication of fused metal shapes on DNA templates. Nano Lett..

[85] Orbach R., Guo W.W., Wang F., Lioubashevski O., Wilner I. (2013). Self-assembly of luminescent Ag nanocluster-functionalized nanowires. Langmuir.

[86] Guo W., Orbach R., Mironi-Harpaz I., Seliktar D., Wilner I. (2013). Fluorescent hydrogels composed of nucleic acid-stabilized silver nanoclusters. Small.

[87] Nilius N., Wallis T.M., Ho W. (2002). Development of one-dimensional band structure in artificial gold chains. Science.

